# Novel PiuC, PirA, and PiuA mutations leading to *in vivo* cefiderocol resistance progression in IMP-16- and KPC-2-producing *Pseudomonas aeruginosa* from a leukemic patient

**DOI:** 10.1128/spectrum.01928-24

**Published:** 2025-01-28

**Authors:** Joaquim Viñes, Sabina Herrera, Andrea Vergara, Ignasi Roca, Jordi Vila, Tommaso Francesco Aiello, José Antonio Martínez, Ana del Río, Carlos Lopera, Carolina Garcia-Vidal, Climent Casals-Pascual, Àlex Soriano, Cristina Pitart

**Affiliations:** 1 Servei de Microbiologia i Parasitologia-CDB, Hospital Clínic de Barcelona 16493 , Barcelona, Spain; 2 Institut de Salut Global (ISGlobal), Barcelona, Spain; 3 Servei Veterinari de Genètica Molecular (SVGM), Facultat de Veterinària, Universitat Autònoma de Barcelona, Bellaterra, Spain; 4 Departament de Malalties Infeccioses, Hospital Clínic de Barcelona 16493 , Barcelona, Spain; 5 Departament de Fonaments Clínics, Facultat de Medicina i Ciències de la Salut, Universitat de Barcelona, Barcelona, Spain; 6 CIBER Enfermedades Infecciosas (CIBERINFEC), Madrid, Spain; Ross University School of Veterinary, Basseterre, Saint Kitts and Nevis

**Keywords:** *Pseudomonas aeruginosa*, carbapenem-resistant, cefiderocol, PiuA, PiuC, PirA, leukemia, Oxford Nanopore

## Abstract

*Pseudomonas aeruginosa* is an opportunistic pathogen capable of causing severe infections in immunocompromised individuals, who often require prolonged antibiotic therapy. The emergence of carbapenemase-producing *P. aeruginosa* has further complicated the management of nosocomial infections, limiting therapeutic options. Cefiderocol has recently emerged as a promising antipseudomonal agent, using the bacterial iron transport system to gain entry into the cell; however, there have been reports of *P. aeruginosa* resistant to cefiderocol. We describe the *in vivo* cefiderocol resistance progression of four consecutive *P. aeruginosa* isolates from one patient with T-cell acute lymphoblastic leukemia. Analysis of potential genes involved in cefiderocol transport resulted in three genes mutated in two resistant isolates. One isolate presented a S116F substitution in PiuC, and the other presented a deletion of 29 amino acids in the signal peptide of PiuA and a STOP substitution in PirA, resulting in the deletion of a piece of the channel. These mutations increased 24- and 64-folds the cefiderocol minimum inhibitory concentration, respectively. The mutations in the aforementioned genes may directly impact siderophore internalization, thereby contributing to an elevation in the MIC of the antibiotic.

**IMPORTANCE:**

Carbapenem-resistant *Pseudomonas aeruginosa* poses a significant challenge due to its broad antibiotic resistance. Cefiderocol is a novel antibiotic aimed at combating infections caused by such organisms. However, if these pathogens develop resistance to this new drug, it hinders treatment efficacy and options. Therefore, it is crucial to identify and describe mutations in the genes involved in the uptake of cefiderocol to find better treatment strategies for patients infected with multidrug-resistant *P. aeruginosa*.

## INTRODUCTION

Nosocomial infections, particularly those caused by *Pseudomonas aeruginosa*, represent a significant clinical challenge in healthcare settings and remains one of the major causes of healthcare-associated infections in Europe (1). *Pseudomonas aeruginosa* is an opportunistic pathogen capable of causing severe infections, especially in immunocompromised individuals, who often require prolonged antibiotic therapy due to their compromised immune systems. In the last decade, there has been a worldwide spread of multidrug-resistant or difficult-to-treat resistance high-risk clones of *Pseudomonas aeruginosa* (2–4). These emerging clones are often associated with the production of carbapenemases, thus also becoming highly resistant to last-resort carbapenem antibiotics (5).

Consequently, there has been a growing reliance on newer antibiotics, such as cefiderocol, designed to target multidrug-resistant gram-negative bacteria. Cefiderocol, a siderophore cephalosporin, uses the bacterial iron transport system to gain entry into the cell, which theoretically enhances its efficacy against resistant strains. However, recent reports (6, 7) indicating resistance to cefiderocol in *P. aeruginosa* are alarming and underscore the urgent need for vigilant antimicrobial stewardship and the development of novel therapeutic strategies. The rapid evolution of antibiotic resistance mechanisms in *P. aeruginosa* requires continuous surveillance and research to mitigate the impact of these formidable nosocomial pathogens on vulnerable patient populations, such as patients with hematological malignancies.

This report aims to highlight the current landscape of *P. aeruginosa* infections in neutropenic patients, the rise of carbapenemase-producing strains, and the implications of emerging resistance to cefiderocol.

## MATERIALS AND METHODS

### Samples, strain identification, cefiderocol susceptibility, and carbapenemase detection

Four *P. aeruginosa* isolates collected in different time points from a leukemic patient admitted to the intensive care unit at the Hospital Clinic of Barcelona in 2023 were included in this study. This patient was a 65-year-old woman native of Cuzco, Peru, who was diagnosed with T-cell acute lymphoblastic leukemia (T-ALL) in June 2022.

Isolates were recovered on selective and differential media from rectal and wound swabs as well as from a urine sample. Routine matrix-assisted laser desorption/ionization time-of-flight mass spectrometry (Bruker Daltonics GmbH & Co. KG, Bremen, Germany) identified the isolates at the species level. Cefiderocol susceptibility testing was performed using diffusion-gradient strips (Liofilchem, Roseto degli Abruzzi, Italy). Interpretation of antibiotic susceptibility and minimum inhibitory concentration (MIC) values followed European Committee on Antimicrobial Susceptibility Testing guidelines and breakpoints for *Pseudomonas* (European Committee on Antimicrobial Susceptibility Testing, 2024 v.14). *P. aeruginosa* American Type Culture Collection 27853 was used for quality control. Production of carbapenemases (KPC, OXA-48 like, IMP, VIM, and NDM) was detected using the lateral flow test NG-CARBA 5 (NG-BIOTECH, France).

### DNA extraction and Nanopore sequencing

ZymoBIOMICS DNA miniprep Kit (Zymo Research, Irvine, USA) was used according to the manufacturer’s protocol to perform DNA extraction, and DNA quality and quantity were assessed with NanoDrop 2000 Spectrophotometer and Quantus Fluorometer with the QuantiFluor dsDNA System (Promega, Madison, USA), respectively.

Nanopore sequencing was performed by using approximately 400 ng of DNA of each isolate to prepare a library with the Rapid Barcoding Sequencing kit (SQK-RBK004; Oxford Nanopore Technologies [ONT], Oxford, UK). The library was sequenced in a MinION FLO-MIN106 v.9.1.4 flow cell (ONT) and the MinION Mk1C device (ONT) for approximately 48 h.

### Genome assembly and analysis

Fast5 reads were live basecalled, and those with a quality of <8 and a length of <200 bp were excluded. Genome assembly was performed with Unicycler v.0.5.0 (8) and polished with Medaka v.1.7.2 (9). Completeness of the polished genomes was assessed with CheckM v.1.2.2 (10) and BUSCO v.5.3.2 (11).

Abricate v.1.0.1 (12), alongside Comprehensive Antibiotic Resistance Database (13) and National Center for Biotechnology Information databases, was used to describe the presence of antibiotic resistance genes, and PlasmidFinder (14) database was used for plasmid replicons. Multilocus sequence type was determined using PubMLST (15). Mutations in different genes were described by aligning the genes of the PAO1 reference genome (GenBank accession number AE004091.2) to the genes within our isolates using BioEdit v.7.2.5 (16), which also allows translation of nucleotide sequences to protein. PiuC and PirA proteins were modeled with AlphaFold2 (17) and SWISS-MODEL (18), respectively.

## RESULTS

### Case history

Here, we present the case of a 65-year-old woman with a history of acute lymphoblastic leukemia pre-B maturation stage, phi-negative, with central nervous system (CNS) involvement that was diagnosed in July 2022 in Peru. She received induction chemotherapy per PETHEMA protocol without L-asparaginase due to her age. She presented with the following complications: neutropenic enterocolitis, *Klebsiella pneumoniae* bloodstream infection, norovirus infection, and COVID-19. At her re-evaluation, she had morphological complete remission, with no blasts in the cerebrospinal fluid. However, she experienced systemic relapse in September 2022 and received rescue therapy with high-dose cytosine arabinoside and mitoxantrone regimen and was kept on maintenance therapy with mercaptopurine. She was first evaluated in our center in search of therapeutic options. She had CNS infiltration and was started on rescue chemotherapy with miniHyperCVAD + rituximab + inotuzumab, together with triple intrathecal therapy.

On 19 February, the patient experienced progressive worsening of respiratory symptoms. A chest X-ray showed consolidation in the mid-right lung field, and the patient was admitted to intensive care with septic shock due to hospital-acquired pneumonia. She was started on piperacillin/tazobactam and teicoplanin. She required invasive ventilation together with vasopressor support. *Stenotrophomonas maltophilia* was isolated on bronchoalveolar lavage. The patient was treated with ceftazidime/avibactam + aztreonam as an alternative regimen to cotrimoxazole to prevent worsening cytopenias. After starting treatment, she presented favorable clinical and radiological improvement and a decrease in inflammatory markers. However, the patient experienced a new febrile episode. A new chest tomography scan was performed, showing worsening bilateral pulmonary consolidations. On 2 March, she was started on cefiderocol, levofloxacin, and isavuconazole, and a new fibrobronchoscopy was performed, isolating <100 CFU/mL of *S. maltophilia*. A catheter tip culture isolated *Enterococcus faecium* and *P. aeruginosa,* so daptomycin was added to the treatment. The patient received a total of 27 days of cefiderocol, and she gradually improved and remained afebrile.

On 5 May, *P. aeruginosa* with KPC and IMP production was isolated in a urine culture without fever, resulting in urinary catheter replacement. A follow-up bone marrow aspiration on 04/12 showed complete morphological remission. In a sacral pressure ulcer swab, multidrug-resistant *P. aeruginosa* (producing KPC + IMP) resistant to cefiderocol was isolated. The ulcer showed no signs of superinfection, and topical dressings and chemical debridement were performed following the recommendations of a specialized pressure ulcer care team.

On 10 May 2023, the patient started having diplopia. As a result, an urgent cerebral angiography magnetic resonance imaging was conducted, revealing leptomeningeal carcinomatosis. With a diagnosis of relapsed pre-B acute lymphoblastic leukemia refractory to second-line treatment in the CNS and a positive minimal residual disease in the bone marrow, a decision was made in consensus with the entire medical team and the family to limit therapeutic efforts.

**Fig 1:**
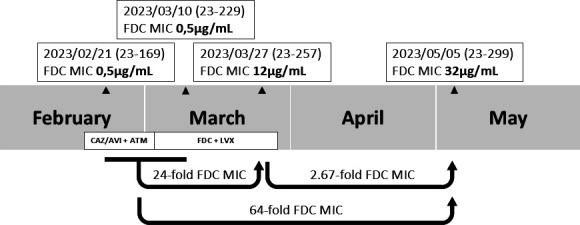
Sample collection date and β-lactam antibiotic therapy timeline. Ceftazidime (CAZ)/avibactam (AVI) plus aztreonam (ATM) treatment was administered from 18 February 2023 to 2 March 2023. Cefiderocol (FDC) plus levofloxacin (LVX) treatment was administered from 2 to 29 March 2023.

Therefore, the patient underwent initial treatment with ceftazidime/avibactam in combination with aztreonam, followed by administration of cefiderocol and levofloxacin. Upon the isolation of the initial strain (23–169, rectal swab, susceptible to cefiderocol with a MIC of 0.5 µg/mL), the first antibiotic treatment had been administered for 2 days. The second strain (23–299, wound, susceptible to cefiderocol with a cefiderocol MIC of 0.5 µg/mL) was obtained after 8 days of cefiderocol treatment, and the third strain (23–257, wound, resistant to cefiderocol with a MIC of 12 µg/mL) was isolated during the continuation of this treatment for a period of 27 days. The fourth *P. aeruginosa* (23–299, urine, resistant to cefiderocol with a cefiderocol MIC of 32 µg/mL) was isolated after the completion of the patient’s entire course of antibiotic treatment (Fig. 1). IMP and KPC carbapenemases were detected in all four isolates.

### Genetic analysis

**Fig 2:**
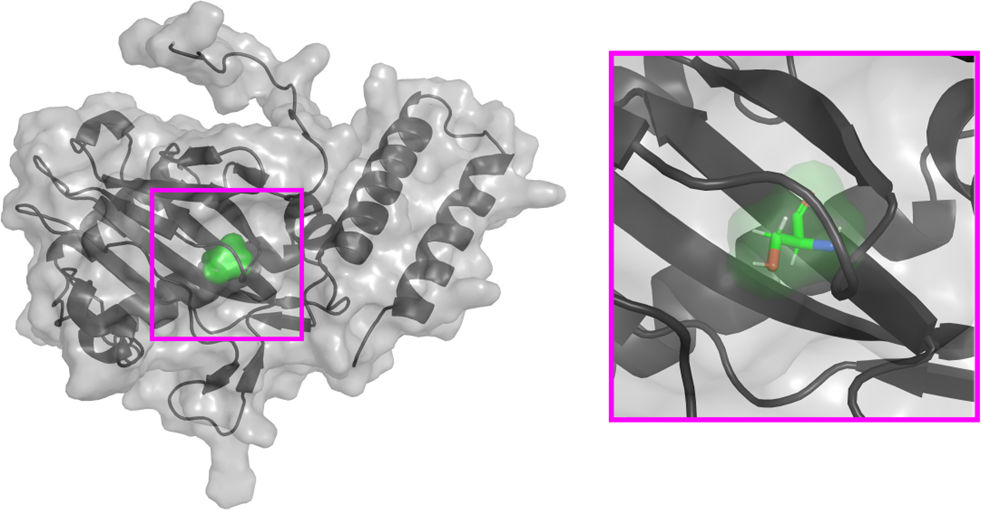
PiuC S116F visualization. The amino acid substitution is located in the center of the β-barrel structure. Created with AlphaFold2.

All isolates belonged to the ST179 and harbored an integron with the *bla*_IMP-16_ gene and an IncP6 plasmid of approximately 34.6 kbp, which carried the *bla*_KPC-2_ carbapenemase gene. For antibiotic susceptibility testing and the antibiotic-resistance gene profile, all isolates presented results consistent with previous findings for isolate 23–169, available from Viñes et al. (5). Forty-eight genes potentially associated with cefiderocol resistance were analyzed (Table S1). Our findings revealed identical mutations in multiple genes shared across all isolates, but only three genes presented mutations exclusively in the two resistant strains: the *piuC* gene in isolate 23–257 and *piuA* and *pirA* genes in isolate 23–299, all of them involved in iron transport. However, shared mutations in concerning genes regarding cefiderocol resistance were also observed. Porin D (443 amino acids, encoded by the *oprD* gene) presented a nonsense mutation at position 49 in the four isolates, and the multidrug resistance operon repressor encoded by the *mexR* gene (147 amino acids) presented a deletion from amino acid 141 to 147. The *piuC* gene encodes the PiuC iron-dependent oxygenase (a TonB-dependent transporter), which features a Fe2OG dioxygenase domain extending from amino acids 78 to 178, and it is involved in the expression of *piuA*. It also presents a conserved β-barrel structure, forming a double-stranded β-helix core fold (19–21). In isolate 23–257, PiuC*,* whose corresponding *piuC* gene is adjacent to *piuA*, presented an S116F amino acid substitution (Fig. 2), located within this latter domain, entailing a switch from an amino acid with polar uncharged side chain (serine) to one with a hydrophobic side chain (phenylalanine), which could be involved in the binding of the siderophore. This isolate showed a 24-fold increase in cefiderocol MIC (12 µg/mL) compared to the first two isolates (0.5 µg/mL).

**Fig 3:**
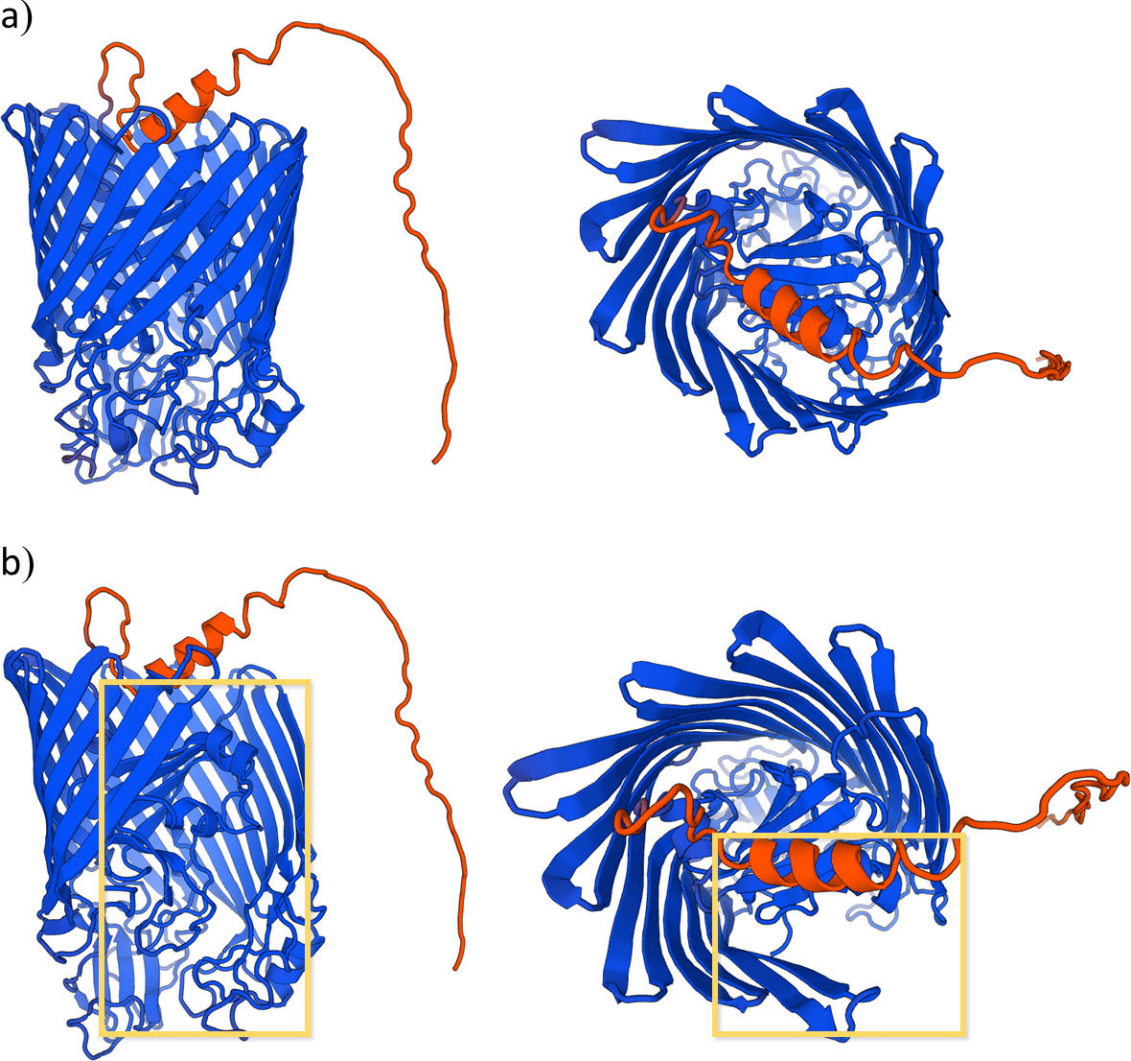
PirA visualization. Structure of PirA from (a) wild-type PAO1 *P. aeruginosa* and (b) 23–299 *P. aeruginosa*. The missing fragment within the β-barrel is shown boxed in yellow. Visualization made with SWISS-MODEL.

The *piuA* and *pirA* genes encode for the PiuA hydroxamate-type ferrisiderophore receptor and the PirA ferric enterobactin receptor (both TonB-dependent receptors), respectively. Both receptors share a common structural framework, comprising two distinct domains: (i) a TonB-dependent receptor plug spanning from amino acids 81 to 176 in PiuA and from amino acids 64 to 178 in PirA; this domain represents a folding subunit functioning as the gate for the channel which, upon ligand binding, undergoes conformational changes and leads to the opening of the channel; and (ii) a domain associated with a TonB-dependent receptor-like β-barrel which shapes the channel. This second domain encompasses amino acids 257–751 in PiuA and amino acids 273–740 in PirA. Additionally, both proteins feature an amino acid signal sequence from amino acids 1 to 35 in PiuA and from amino acids 1 to 28 in PirA (22, 23). In isolate 23–299, the *piuA* gene presented a deletion of 87 nucleotides, which was translated to a deletion of 29 amino acids (28–56, both included), resulting in the removal of a segment of the signal sequence. Moreover, the *pirA* gene presented a nonsense mutation C1908G translated into Y636STOP, which induces premature termination of the protein translation and alters the structure of the β-barrel (Fig. 3). This isolate showed a 64-fold increase in cefiderocol MIC (32 µg/mL) compared to the first two isolates (0.5 µg/mL) and a 2.67-fold increase compared to the 22–257 isolate (12 µg/mL).

## DISCUSSION

In this report, we present the in-patient evolution of cefiderocol resistance observed in four consecutive *P. aeruginosa* isolates recovered from a 65-year-old woman diagnosed with T-ALL and treated with cefiderocol for 27 days.

Cefiderocol, an antibiotic resulting from the fusion of a siderophore and a cephalosporine sharing similarities with cefepime and ceftazidime, has emerged as a promising candidate for the treatment of multidrug-resistant gram-negative bacteria, attributed to its demonstrated efficacy against a diverse spectrum of pathogens, including Enterobacterales, *Acinetobacter baumannii*, and *P. aeruginosa* resistant to carbapenems, resistant to β-lactam/β-lactamase inhibitor combinations, as well as resistant to polymyxins. Nevertheless, several reviews (24–26) have outlined numerous cefiderocol resistance mechanisms, which can be summarized as

1. Carriage of certain β-lactamases, which can be correlated to higher cefiderocol MICs, such as in *Escherichia coli* with different copies of *bla_NDM-5_* genes (27).
2. Defects in cell permeability and/or increased efflux of cefiderocol due to mutations affecting porins or siderophore receptors, such as OprD (28) and PiuA/PirA (29, 30) in *P. aeruginosa*, and overexpression of efflux pumps, such as MexAB-OprM, also in *P. aeruginosa* (30).
3. Target modifications and presence of other genes with potential involvement in cefiderocol resistance, such as modification of PBP-3, which is the target of cefiderocol, shown in *E. coli* and *A. baumannii* (31, 32).

Nonetheless, it has been proposed that for a particular isolate to develop resistance to cefiderocol, it must exhibit a combination of some of the above (24, 25). Moreover, it has been previously reported that treatment with a combination of a cephalosporine and a β-lactamase inhibitor (such as ceftolozane plus tazobactam or ceftazidime plus avibactam) in absence of cefiderocol treatment may contribute to the emergence of cefiderocol resistance. For instance, the emergence of mutations within the YSN region of AmpC in *P. aeruginosa* potentially results in reduced susceptibility to cefiderocol (33–35).

In this study, we compared the sequence evolution of 48 genes from four different isolates recovered from a single patient during the course of prolonged cefiderocol treatment (27 days) and compared them against the *P. aeruginosa* PAO1 reference genome. Our analysis included genes encoding for TonB-dependent receptor proteins involved in siderophore transport, genes involved in iron uptake, chromosomal β-lactamases, and penicillin-binding proteins, among others, all of which are potentially involved in cefiderocol resistance (Table S1). Mutations in porine D (*oprD*) and the multidrug resistance operon repressor (*mexR*) were described across the four isolates, and previous studies have shown that truncations in the *oprD* gene can increase cefiderocol MIC by 2-fold (0.125–0.25 mg/L) (30) and 16-fold (0.25–4.0 mg/L) (28). Additionally, a transposon insertion in the *mexR* gene can double the MIC (0.125–0.25 mg/L) (30). Various MexR amino acid substitutions, such as A66V and L57D, have been associated with four- and eightfold increases in MIC, respectively (34). Furthermore, the D89E mutation in MexR was linked to a fourfold increase in MIC (2–8 mg/L) in progressive *in vivo* samples (36).

However, despite the mutations in the *oprD* and *mexR* genes, the only mutations related to resistant isolates in this study were a S116F substitution in the *piuC* gene in isolate 23–257 and an 87-nucleotide deletion in *piuA* gene and Y636STOP nonsense mutation in PirA in isolate 23–299. Siderophore transporters (also known as TonB-dependent transporters) are located in the outer membrane of the bacterial cell. Their role involves capturing the siderophore-iron complex and facilitating its internalization into the periplasm (37, 38). Consequently, upon translation, the protein has to reach its final location, for which the signal sequence promotes the secretion of the protein to the outer membrane and subsequent insertion (39, 40). Therefore, a deletion in the signal sequence of PiuA in isolate 23–299 may lead to the receptor’s absence in the outer membrane, resulting in a lower internalization of the siderophore-conjugated antibiotic. Conversely, the deletion observed in PirA from isolate 23–299 influences the structural configuration of the channel, as illustrated in Fig. 3. This alteration has the potential to impact the overall integrity and functionality of the protein, resulting in a reduced internalization of the antibiotic, as previously noted for PiuA. To support the potential role of mutations in PirA and PiuA proteins in cefiderocol resistance, prior studies have been conducted that linked them to lower susceptibility to siderophore-conjugated antibiotics. As described by van Delden et al. (41) and Ito et al. (30), deficiency of the PiuA receptor in some strains led to a decrease in the siderophore-antibiotic susceptibility, raising by 32- and 16-folds the MIC of the antibiotic tested, respectively. In another study performed by Luscher et al. (29), the absence of PiuA led to a 16-fold increase of the siderophore conjugate and a 32-fold increase when combined with a deletion in *pirA*. Other mutations in *piuA* and *pirA* genes, such as insertions, deletions, and frameshifts, did also increase the MIC to siderophore-conjugate molecules in *P. aeruginosa*, as described by Kim et al. (42). Regarding the PiuC S116F amino acid substitution in isolate 23–257, it has been previously discussed (43) that the influence of amino acid polarity and hydrophobicity on channel proteins is noteworthy. The research suggests that hydrophobic residues interact unfavorably with water, potentially creating an energetic impediment to ion conduction, a phenomenon referred to as hydrophobic gating. Hence, the substitution of a polar amino acid with a hydrophobic one in PiuC in isolate 23–257 may disrupt the interaction between the ion and the channel, thereby impeding further processes such as expression of *piuA* (21). Additionally, *P. aeruginosa* PiuC, which presented frameshift mutations, downregulations, and indels, showed increased MICs against siderophore-conjugated antibiotics (21, 41, 42, 44).

Of note, while cefiderocol resistance in isolate 23–257 was attributed to a mutation within the *piuC* gene, such mutation was not carried over by the latest isolate 23–299 that nevertheless showed higher cefiderocol MIC values through mutations in both the *piuA* and *pirA* genes. Therefore, development of cefiderocol resistance under selective pressure seemed to have occurred as two independent events within the same patient and on the same strain.

In conclusion, we describe the *in vivo* emergence of cefiderocol resistance in *P. aeruginosa* isolated from a patient with T-ALL who underwent treatment with ceftazidime-avibactam and cefiderocol. We identified several mutations among the analyzed genes shared by the four isolates, including mutations in *oprD* and *mexR* genes that may affect cefiderocol’s MIC. Only three genes presented mutations specifically in those isolates resistant to cefiderocol, namely, *piuC*, *piuA*, and *pirA*. The mutations in the three aforementioned genes may directly impact siderophore internalization, thereby contributing to an elevation in the MIC of the antibiotic. Future steps will include experimentally demonstrating the effect of the mutations on the MIC of *P. aeruginosa*, as this study only identified them *in silico*.

## Data Availability

Nanopore FastQ read files have been submitted to Sequence Read Archive from the National Center for Biotechnology Information under BioProject PRJNA984723. The GenBank accession numbers for the genome assemblies are as follows: 23–169 JAUAWL000000000.1, 23–229 JAXASH000000000, 23–257 JAXASG000000000, and 23–299 JAXASF000000000.

## SUPPLEMENTAL MATERIAL

Table S1 spectrum.01928-24-s0001.xlsx Mutations present in genes of interest that may be involved in cefiderocol resistance.

ASM does not own the copyrights to Supplemental Material that may be linked to, or accessed through, an article. The authors have granted ASM a non-exclusive, world-wide license to publish the Supplemental Material files. Please contact the corresponding author directly for reuse.
